# Long non-coding RNA GAS5 aggravates myocardial depression in mice with sepsis via the microRNA-449b/HMGB1 axis and the NF-κB signaling pathway

**DOI:** 10.1042/BSR20201738

**Published:** 2021-04-09

**Authors:** Hongfeng Gao, Huijing Ma, Min Gao, Aichun Chen, Shujuan Zha, Jixi Yan

**Affiliations:** 1 Department of Emergency Medicine, Wuhan Wuchang Hospital, Wuchang Hospital Affiliated to Wuhan University of Science and Technology, Wuhan 430000, Hubei, P.R. China; 2 Department of Image, Wuhan Children’s Hospital (Wuhan Maternal and Child Healthcare Hospital), Tongji Medical College, Huazhong University of Science and Technology, Wuhan 430000, Hubei, P.R. China; 3 Department of Anesthesia, Hanyang Hospital Affiliated to Wuhan University of Science and Technology, Wuhan 430000, Hubei, P.R. China

**Keywords:** HMGB1, Long-noncoding RNA GAS5, microRNA-449b, Myocardial depression, Myocardial injury, Sepsis

## Abstract

Sepsis is a common cause of deaths of patients in intensive care unit. The study aims to figure out the role of long non-coding RNA (lncRNA) GAS5 in the myocardial depression in mice with sepsis. Cecal ligation and puncture (CLP) was applied to induce sepsis in mice, and then the heart function, myocardium structure, and the inflammatory response were evaluated. Differentially expressed lncRNAs in mice with sepsis were identified. Then gain- and loss-of-functions of GAS5 were performed in mice to evaluate its role in mouse myocardial depression. The lncRNA-associated microRNA (miRNA)–mRNA network was figured out via an integrative prediction and detection. Myocardial injury was observed by overexpression of high-mobility group box 1 (HMGB1) in septic mice with knockdown of GAS5 expression. Activity of NF-κB signaling was evaluated, and NF-κB inhibition was induced in mice with sepsis and overexpression of GAS5. Collectively, CLP resulted in myocardial depression and injury, and increased inflammation in mice. GAS5 was highly expressed in septic mice. GAS5 inhibition reduced myocardial depression, myocardial injury and inflammation responses in septic mice. GAS5 was identified to bind with miR-449b and to elevate HMGB1 expression, thus activating the NF-κB signaling. HMGB1 overexpression or NF-κB inactivation reduced the GAS5-induced myocardial depression and inflammation in septic mice. Our study suggested that GAS5 might promote sepsis-induced myocardial depression via the miR-449b/HMGB1 axis and the following NF-κB activation.

## Introduction

Sepsis is a deadly organ functional impairment resulting from an imbalance in the body’s reaction to infection [1]. Sepsis takes up 2–6% of all hospital inpatients and accounts for up to 15% of in-hospital mortality, and the mortality is even higher when it is associated with hypotension or hypoperfusion (namely septic shock) [2]. In both developing and developed countries, sepsis is a usual cause of death and brings considerable healthcare burdens [3]. Despite advanced comprehension in the mechanisms involved, severe sepsis still takes up a rising number of deaths in particularly ill patients [4]. Notably, sepsis usually leads to myocardial dysfunction which is usually defined as sepsis-induced myocardial depression or heart dysfunction, featured with damaged myocardial contractility as well as impaired ejection fraction [5]. Approximately 40–50% of patients with septic shock develop different degrees of myocardial depression or dysfunction on their left ventricular ejection fraction, and the mortality of these patients is up to 70% [6,7]. Hereby, identifying molecular mechanisms involving in the sepsis-induced myocardial depression and injury is of great necessity.

Long non-coding RNAs (lncRNAs) and microRNAs (miRNAs) are two main classes of non-protein-coding transcripts that mediate essential cellular processes through multiple mechanisms [8]. LncRNAs are over 200 nucleotides in length and are involved in diverse physiological and pathological processes of multiple diseases including sepsis [9]. Through literature review [5,10], we noticed that lncRNAs differentially expressed play an important role in septic mice. A recent study demonstrated that many lncRNAs are expressed differently in the sepsis and control groups and play a critical role in the process of myocardial depression [5]. GAS5 may be a new target for the treatment of myocardial injury [11–13]. Importantly, the competing endogenous RNA (ceRNA) networks have aroused wide concerns recently, through which the lncRNAs can regulate gene expression via competitively binding to miRNAs through miRNA response elements [14,15]. miRNAs are approximately 21 nucleotides in length and work as guide molecules that induce mRNA degradation or block protein translation of the target genes [16]. Likewise, miRNAs have been suggested to be closely correlated with the diagnosis and staging of sepsis, and they may exert key functions in mediating the sepsis outcome [17]. Moreover, many miRNAs have been suggested to play key regulating roles in myocardial depression or cardiac dysfunction induced by sepsis [18]. Interestingly, several lncRNA–miRNA–mRNA interactions have been found functioning in sepsis [19,20]. In light of these findings, this study hypothesized that there would be potential ceRNA networks involved in the sepsis-induced myocardial depression. Herein, we identified one of the differentially expressed lncRNAs, GAS5 after sepsis, and then measured whether GAS5 participates in the myocardial depression in mouse models, and had the further mechanisms explored.

## Materials and methods

### Mouse models of sepsis and grouping

C57BL/6 mice (25–30 g) purchased from Southern Medical University (SYXK (Guangdong) 2016-0167) were raised in cages in a temperature-controllable room under a 12-h light/dark cycle, with free access to food and water. Twenty-four mice were randomly selected and allocated into sham group (*n*=12) and cecal ligation and puncture (CLP) group (*n*=12), to assess the survival rate of mice within 48 h following surgery. Mice were sent back to the cages with water and food supply, and the survival rate of mice after CLP was measured every 2 h for a total of 48 h. The survived mice underwent CLP were intraperitoneally injected with overdose of pentobarbital sodium (800 mg/kg) (Sinopharm Chemical Reagent Co., Ltd., Shanghai, China) for euthanasia to reduce their suffering.

In the following experiments, another 120 mice were used, among which 12 mice were subjected to sham surgery while the rest mice underwent CLP and assigned into nine groups (*n*=12 each group): CLP/Sepsis group (septic mouse models); Sepsis + LV-GAS5-shRNA group [mice were injected with lentiviral vectors (LV)-GAS5-shRNA (5 × 10^7^ infectious lentivirus particles per mouse) through the caudal vein 1 week before CLP and injected again 3 days after the initial injection [21,22]]; Sepsis + LV-GAS5 group (mice were injected with GAS5 as the same procedures mentioned above); Sepsis + PBS group (mice were injected with phosphate buffer saline for control); Sepsis + LV-NC group (mice were injected with LV-negative control); LV-GAS5 + PDTC group (PDTC, pyrrolidine dithiocarbamate, a nuclear factor-κ B (NF-κB) inhibitor, BioVision, Mountain View, CA, U.S.A.; mice were injected with LV-GAS5 as mentioned above, and further intraperitoneally injected with 100 mg/kg PDTC 2 h before CLP [23]); LV-GAS5 + Vehicle group [mice were treated as the LV-GAS5 + PDTC group but had the PDTC replaced by same volume of vehicle dimethyl sulfoxide (6.7 ml/kg, Solarbio Science & Technology, Beijing, China)], LV-GAS5 + oe-NC group (mice were injected with GAS5 and negative control of high-mobility group box 1 (HMGB1) overexpression lentivirus by tail vein injection of 5 × 10^7^ infectious lentivirus particles per mouse), and LV-GAS5 + oe-HMGB1 group (mice were injected with GAS5 and HMGB1 overexpression lentivirus by tail vein injection of 5 × 10^7^ infectious lentivirus particles per mouse). The LV-GAS5-shRNA (GAS5 depletion), LV-GAS5 (GAS-5 overexpression) and HMGB1 overexpression lentivirus (oe-HMGB1) and the control (oe-NC) were synthesized by GenePharma Co, Ltd., (Shanghai, China). The mouse hearts were monitored 12 h after CLP, and the myocardial homogenate and serum samples of six mice from each group were collected for experiments, while the rest of the six mice were used for Hematoxylin and Eosin (HE) staining.

CLP was applied for establishment of mouse models of sepsis. The procedures were performed as previously reported [24]. In brief, a midline laparotomy was introduced with a 3-cm dissection for cecum exposure. The cecum was ligated with 4-0 silk ligatures at the position designated for the high severity grade and punctured at two sites apart by 1 cm with a 20-gauge needle. The cecum was then pressed gently to extrude a small droplet of feces from the holes and then returned to the abdominal cavity. After the laparotomy was over, all mice were resuscitated via subcutaneous injection of pre-warmed normal saline (37°C, 50 ml/kg). The sham operation was performed as the same procedures without ligation and puncturation on the cecum.

### Monitoring of heart function

The heart function was monitored as previously suggested [25]. All mice were anesthetized via intraperitoneal administration of pentobarbital sodium 12 h after CLP, and the adequate anesthesia was confirmed by impaired contractile response of mice after stimulations on lower limbs. Next, the right carotid artery was exposed and inserted with a catheter, and the changes of mean arterial pressure (MAP) of mice were determined via a multichannel data acquisition and processing system (RM6240BD, Chengdu Instrumeny Factory, Chengdu, Sichuan, China). Then the catheter was sent into the left ventricle through the right carotid artery, by which the changes of left ventricular systolic pressure (LVSP), maximal rate of the increase in left ventricular pressure (+*dp/dt*max), and maximal rate of the decrease in left ventricular pressure (−*dp/dt*max) were measured.

### HE staining

As followed by the standardized HE staining procedures, myocardial tissues from mice were fixed with 4% paraformaldehyde, and then embedded with paraffin, sectioned, and stained with HE. Then the structure of the tissues was observed under a microscope.

### Immunofluorescence

The myocardial tissue fixed with 4% paraformaldehyde was embedded with Optical Coherence Tomography (OCT) frozen embedding agent, and the frozen sections were stained with immunofluorescence staining. The sections were incubated with primary antibody NF-κB p65 (1:50; ab16502) at room temperature for 2 h, and then with the second antibody IgG for 1 h (1:200; ab150077). The nuclei were stained with DAPI for 20 min at room temperature without light exposure and observed under the fluorescence microscope.

### Reverse transcription quantitative polymerase chain reaction

Total RNA from myocardial tissue homogenate was extracted using TRIzol Reagent (Invitrogen Inc., Carlsbad, CA, U.S.A.) as per the manufacturer’s protocols, and the RNA was reverse transcribed into cDNA using a One-Step PrimeScript miRNA cDNA Synthesis kit (Takara Biotechnology Ltd., Dalian, China) or a PrimeScript™ RT Master Mix (Takara). Reverse transcription quantitative polymerase chain reaction (RT-qPCR) was performed as per the protocol of the SYBR Premix Ex Taq (Takara) with cDNA as the template, and conducted on a Roche LightCycler 480 (Roche Ltd, Basel, Switzerland). The primers are shown in Table 1. Results were exhibited as arbitrary units as compared with the mRNA expression of U6.

**Table 1:** Primer sequences for RT-qPCR

Primer	Sequence (5′–3′)
miR-449b	F: GGCAGGCAGTGTTGTTAGCTGG
	R: GTGCAGGGTCCGAGGT
U6	F: ATTGGAACGATACAGAGAAGAT
	R: GGAACGCTTCACGAATTTG
HMGB1	F: GCGAGCATCCTGGCTTATC
	R: TTCAGCTTGGCAGCTTTCT
GAPDH	F: CATTGCTGACAGGATGCAGA
	R: CTGCTGGAAGGTGGACAGTGA

Abbreviations: F, forward; GAPDH, glyceraldehyde-3-phosphate dehydrogenase; R, reverse.

### Western blot analysis

Total proteins from myocardial homogenate were extracted using a protein extraction kit (Beyotime Institute of Biotechnology, Inc., Shanghai, China), and then the protein concentration was determined with a bicinchoninic acid kit (Nanjing Jiancheng Bioengineering Institute, Nanjing, Jiangsu, China). Next, the proteins were loaded for electrophoresis and the protein gels were transferred on to polyvinylidene fluoride membranes (Millipore, Bedford, MA, U.S.A.). The membranes were sealed with Tris-buffered saline (5% skim milk) containing 0.1% Tween-20 at room temperature for 1 h, and then incubated with the primary antibodies p-p65 (1:2000, ab86299, Abcam), NF-κB p65 (1:1000, #8242, Cell Signaling, Beverly, Massachusetts, U.S.A.), HMGB1 (1:1000, ab79823, Abcam) and glyceraldehyde-3-phosphate dehydrogenase (GAPDH; 1:1000, ab181602, Abcam) at 4°C overnight. Next, the membranes were further incubated with secondary antibody horseradish peroxidase-conjugated goat anti-rabbit immunoglobulin G (1:5000, ab205718, Abcam) to assess the immunoreactivity of the proteins. The density of the protein bands was analyzed using Image-Pro Plus version 6.0 (Media Cybernetics, Silver Spring, MD, U.S.A.) [26].

### Enzyme-linked immunosorbent assay

Myocardial homogenate was centrifuged and the supernatant was collected. Then the levels of tumor necrosis factor-α (TNF-α, PRTA00), interleukin (IL)-6 (PR6000B) and IL-1β (PRLB00) were measured in line with the instructions of enzyme-linked immunosorbent assay (ELISA) kits (R&D Systems, Minneapolis, MN, U.S.A.). Meanwhile, the contents of creatine kinase-MB (CK-MB, E006-1-1) and cardiac Troponin I (cTnI, H149-2) in serum were measured using the corresponding kits (Nanjing Jiancheng).

### Nitric oxide content assessment

The nitric oxide (NO) content in mouse myocardial tissues was determined using an NO assay kit (nitrate reductase method) (A012-1-1, Nanjing Jiancheng).

### Microarray analysis for lncRNAs

Microarray analysis for lncRNAs was performed by Shanghai Biotechnology Corporation (Shanghai, China). Total RNA from mouse myocardium was extracted using TRIzol and analyzed using Arraystar mouse lncRNA Array v2.0. The background analysis data were excluded, and the signals were standardized by locally weighted scatter plot smoothing (LOWESS) using a LOWESS wave filter.

### Dual luciferase reporter gene assay

A computer-based program Jefferson (https://cm.jefferson.edu/rna22/Precomputed/) was applied to measure the target binding sites of lncRNAGAS5, which suggested that miR-449b could specifically bind to GAS5. Besides, prediction on TargetScan (http://www.targetscan.org/vert_72/) suggested that miR-449b could directly bind to the 3′-untranslated region of HMGB1. The wildtype GAS5 and HMGB1 3′UTR were amplified and cloned to the pmiR-GLO reporter vector (Promega, U.S.A.), respectively. The mutants of GAS5 (5′-ACUGCUU-3′→5′-UGACGUA-3′) and HMGB1 3′UTR (5′-ACUGCCA-3′→5′-UGACGGA-3′) were constructed using the site-directed mutagenesis kit (YEASEN, Shanghai, China) according to the instructions. Well-constructed vectors were co-transfected with either mimic-NC or mimic-miR-449b into HEK293T cells (Shanghai Institute of Biochemistry and Cell Biology, Chinese Academic of Science, Shanghai, China). The relative luciferase activity was detected 48 h later. The experiment was performed in triplicate. The mimic-miR-449b and mimic-NC were synthesized by GenePharma Co, Ltd.

### Statistical analysis

The SPSS 21.0 (IBM Corp. Armonk, NY, U.S.A.) was used for data analysis, and data images were drawn with GraphPad Prism 8.0 (GraphPad Software, San Diego, CA, U.S.A.). Kolmogorov–Smirnov tested the data were in normal distribution and presented as mean ± standard deviation (SD). Differences between every two groups were evaluated using the independent sample *t* test, while differences among multiple groups were compared using one-way analysis of variance (ANOVA) or two-way ANOVA. Tukey’s multiple comparisons test was used for the pairwise comparisons after ANOVA. The *P*-value was obtained from a two-tailed test, and *P*<0.05 was considered as statistically significant difference, while *P*<0.01 was considered as great statistically significant difference.

## Results

### Sepsis induces myocardial depression/injury in mice

**Figure 1:**
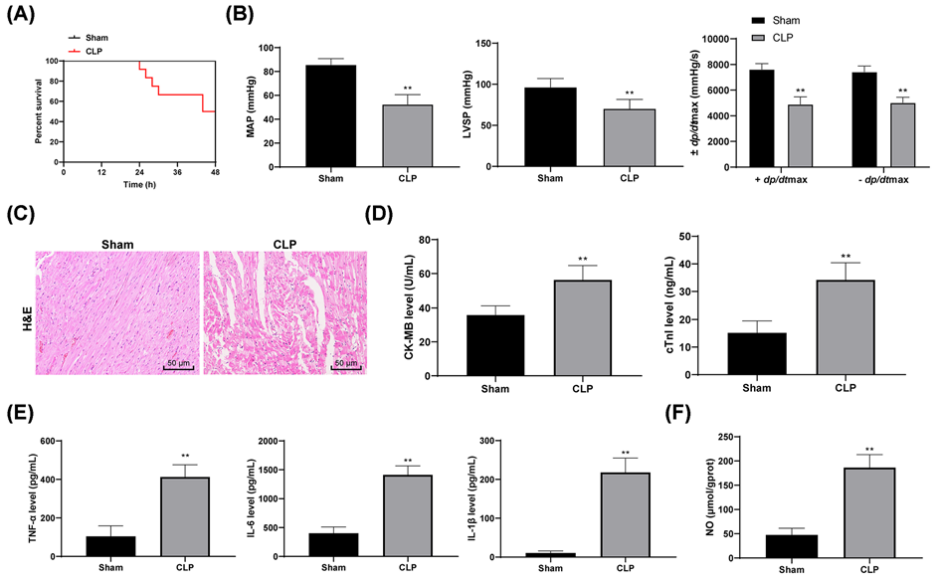
Sepsis induces myocardial depression/injury in mice (**A**) Diagram for the survival rate of mice within 48 h following CLP surgery, to each group; (**B**) evaluation of the MAP, LVSP and ±dp/dtmax values; (**C**) structure of myocardium of mice observed via HE staining; (**D**) levels of cTnI and CK-MB in mouse serum measured using ELISA; (**E**) levels of inflammatory factors TNF-α, IL-6 and IL-1β in mouse myocardial tissues measured using ELISA; (**F**) NO content in mouse myocardial tissues evaluated using nitrite/nitrate kit. In panels (A,B), *n*=12, while in panels (C–E) *n*=6; date are exhibited as mean ± SD; differences between every two groups were analyzed using the independent sample *t* test, while the differences among multiple groups were analyzed via two-way ANOVA; **, *P*<0.01.

Mouse models of sepsis were established via introducing CLP in mice. The survival rate of mice was evaluated within 48 h following surgery. Mice underwent CLP began to die 24 h after surgery and the survival rate was 50% at 48 h after surgery, while those underwent sham surgery were all survived (Figure 1A). Meanwhile, the heart function of mice was measured 12 h after CLP, which suggested the MAP, LVSP and ±*dp/dt*max values were decreased in mice underwent CLP compared with the sham-operated mice (all *P*<0.01) (Figure 1B), indicating that the cardiac function of septic shock mice was inhibited. The results of HE staining showed that the myocardium of the sham-operated mice was arranged orderly and the nucleus was clear. However, after CLP surgery, the myocardial structure was damaged, and the myocardium was obviously swollen and disordered, and there was inflammatory cell infiltration (Figure 1C). We next found that the contents of myocardial injury markers cTnI and CK-MB in serum were increased in mice following CLP (all *P*<0.01) (Figure 1D), and the levels of TNF-α, IL-6 and IL-1β, and the NO content in myocardial tissues were elevated as well (all *P*<0.01) (Figure 1E,F). These results suggested that myocardial injury occurred in septic mice.

### Knockdown of GAS5 alleviates sepsis-induced myocardial depression/injury in mice

**Figure 2:**
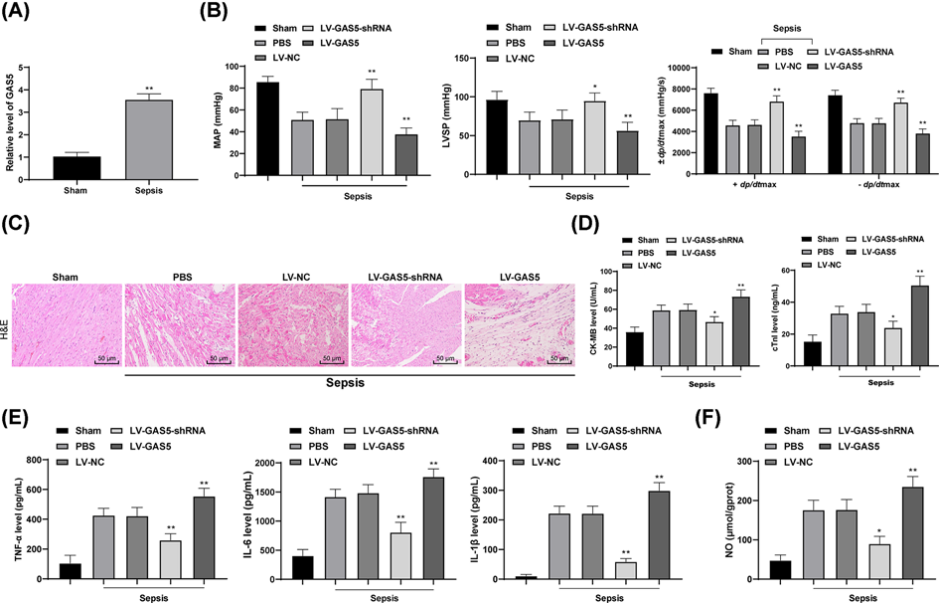
Knockdown of GAS5 alleviates sepsis-induced myocardial depression/injury in mice (**A**) GAS5 expression in mouse myocardium detected using RT-qPCR; (**B**) evaluation of the MAP, LVSP and ±dp/dtmax values of mice following GAS5 interference; (**C**) structure of myocardium of mice observed via HE staining; (**D**) levels of cTnI and CK-MB in mouse serum measured using ELISA; (**E**) levels of inflammatory factors TNF-α, IL-6 and IL-1β in mouse myocardial tissues measured using ELISA; (**F**) NO content in mouse myocardial tissues evaluated using nitrite/nitrate kit. In panels (B–F) mice with sepsis were transfected with LV-GAS5-shRNA or LV-GAS5 to have depleted or overexpressed GAS5 expression; in panel (B) *n*=12, while in the rest of the panels, *n*=6; data are expressed as mean ± SD; data in panel (B) were analyzed using the independent sample *t* test, while data in panels (C–G) were analyzed using one-way ANOVA and Tukey’s multiple comparisons test; *, *P*<0.05, **, *P*<0.01, compared with the sepsis + LV-NC group.

Through literature review [5,10], we noticed that lncRNAs differentially expressed play an important role in septic mice, and GAS5 may be a new target for the treatment of myocardial injury [11,13]. The RT-qPCR identified that GAS5 is highly expressed in mice with sepsis (*P*<0.01) (Figure 2A). Next, artificial inhibition or overexpression of GAS5 was introduced via transfecting LV-GAS5-shRNA or LV-GAS5 vectors into the myocardial tissues of rats with sepsis. Then we found the MAP, LVSP and ±*dp/dt*max values were evidently up-regulated following GAS5 inhibition, indicating heart function of mice with sepsis was partly recovered, and the myocardial depression/injury was reduced. Reasonably, up-regulation of GAS5 led to cardiac dysfunction and aggravated injury in mice (all *P*<0.01) (Figure 2B,C). Meanwhile, GAS5 inhibition decreased the contents of cTnI and CK-MB in mouse serum (all *P*<0.01) (Figure 2D), as well as the levels of TNF-α, IL-6 and IL-1β (all *P*<0.01) (Figure 2E), and the NO content in myocardial tissues (*P*<0.01) (Figure 2F), while overexpression of GAS5 exacerbated the inflammation and myocardial depression/injury in mice with sepsis (all *P*<0.05) (Figure 2D-F). It is suggested that the increase in GAS5 expression can aggravate the pro-inflammatory reaction and myocardial injury.

### GAS5 regulates HMGB1 via interacting with miR-449b

**Figure 3:**
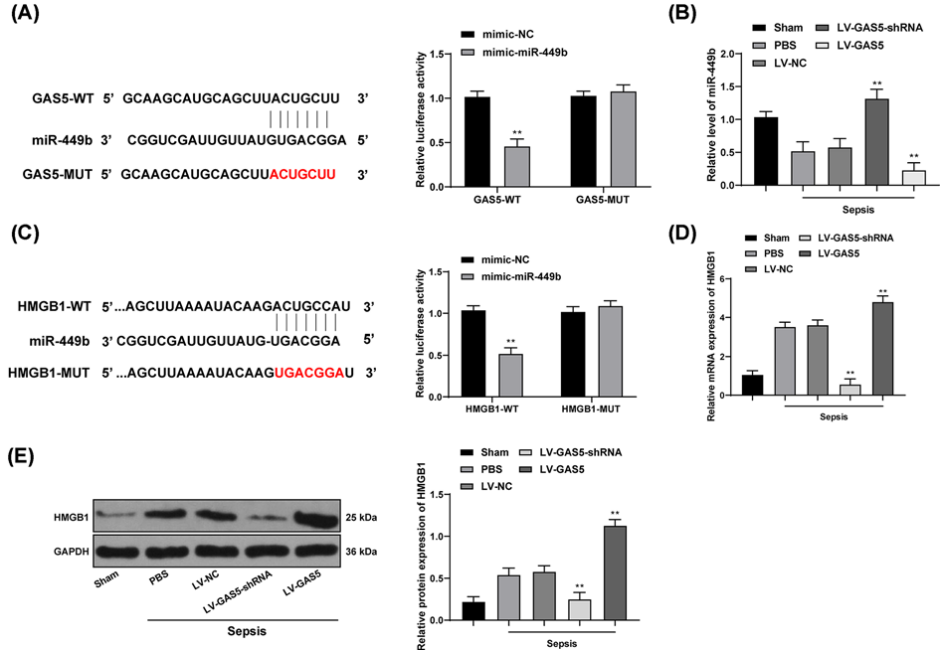
GAS5 regulates HMGB1 via interacting with miR-449b (**A**) Binding site between GAS5 and miR-449b predicted on Jefferson (https://cm.jefferson.edu/rna22/Interactive/) and identified with dual luciferase reporter gene assay; (**B**) miR-449b expression in mouse myocardium measured using RT-qPCR; (**C**) binding site between miR-449b and HMGB1 predicted on TargetScan (http://www.targetscan.org/vert_72/) and identified with dual luciferase reporter gene assay; (**D,E**) mRNA expression (D) and protein level (E) of HMGB1 in mouse myocardium detected using RT-qPCR and Western blot analysis, respectively. To each group, *n*=6; data were expressed as mean ± SD; in panels (A,C), data were analyzed using two-way ANOVA, while in the rest of the panels, data were analyzed using one-way ANOVA, and Tukey’s multiple comparisons test was applied for post hoc test after ANOVA; *, *P*<0.05, **, *P*<0.01, compared with the sepsis + LV-NC group.

The Jefferson system predicted that GAS5 could directly bind to miR-449b, which was validated through the dual luciferase reporter gene assay (*P*<0.01) (Figure 3A). Next, miR-449b expression was further measured using RT-qPCR, which suggested that miR-449b expression was reduced in mice with sepsis, while LV-GAS5-shRNA transfection partly recovered miR-449b expression (all *P*<0.01) (Figure 3B). Meanwhile, we found miR-449b could bind to the downstream HMGB1 via bioinformatics prediction and luciferase assays (all *P*<0.01) (Figure 3C), and the mRNA and protein levels of HMGB1 were elevated in mice with sepsis. LV-GAS5-shRNA transfection decreased HMGB1 expression in mice, which was partly reversed by LV-GAS5 (all *P*<0.05) (Figure 3D,E).

### Overexpression of HMGB1 attenuates the protective effect of GAS5 knockdown on myocardium in septic mice

**Figure 4:**
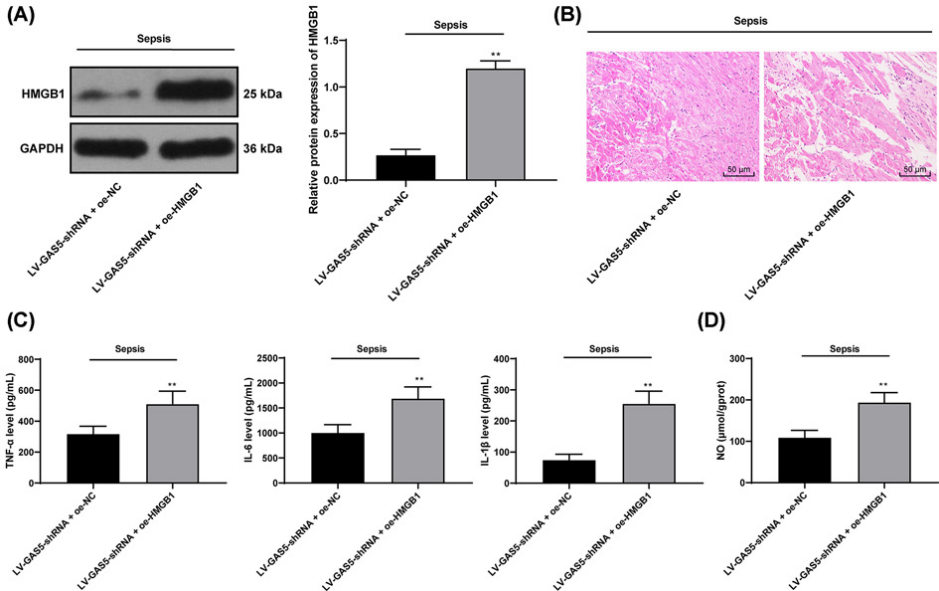
Overexpression of HMGB1 attenuates the myocardial protective effect of GAS5 knockdown on septic mice (**A**) Western blot was used to detect the effect of overexpression of HMGB1 on the expression of HMGB1 in septic mice. (**B**) HE staining was used to observe the effect of HMGB1 overexpression on myocardial pathological damage in septic mice. (**C**) The effects of HMGB1 overexpression on the levels of TNF-α, IL-6 and IL-1β in septic mice were detected by ELISA. (**D**) Nitrite/nitrate kit was used to detect the content of NO in myocardium of septic mice after knockdown of GAS5 and HMGB1 overexpression. The sample size of all groups was *n*=6, and the results were expressed by means ± SD. The differences between groups were analyzed by independent sample *t* test, **P<0.01 compared with LV-GAS5 + oe-NC group.

From the above results, we found that GAS5 may regulate the expression of HMGB1 through miR-449b. Therefore, we set up a rescue experiment to overexpress HMGB1 (oe-HMGB1) in septic mice with GAS5 knockdown (Figure 4A, *P*<0.01). Compared with the septic mice with knockdown of GAS5, HMGB1 overexpression reduced myocardial injury and increased inflammatory cell infiltration (Figure 4B). Meanwhile, the levels of TNF-α, IL-6, IL-1β and NO in myocardium were significantly increased relative to LV-GAS5 + oe-NC group (Figure 4C,D, all *P*<0.01).

### Knockdown of GAS5 inactivates the HMGB1/NF-κB signaling pathway in mice with sepsis

**Figure 5:**
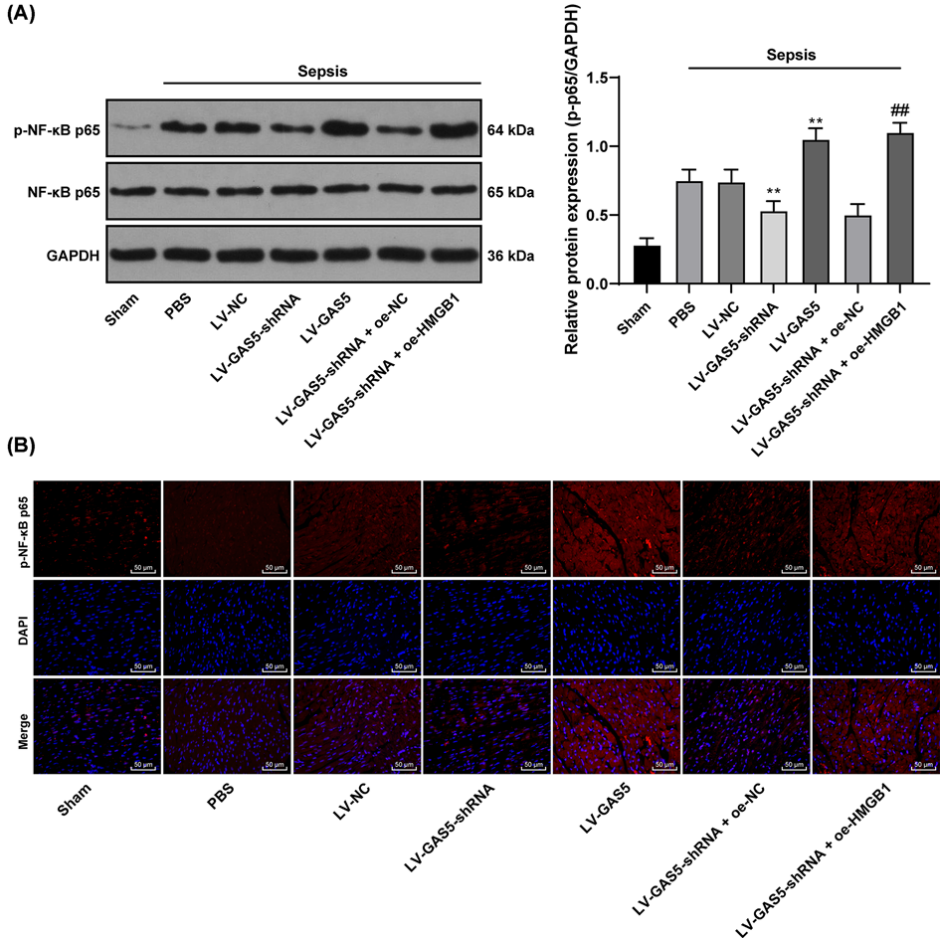
Knockdown of GAS5 inactivates the HMGB1/NF-κB pathway in mice with sepsis LV-GAS5-shRNA or LV-GAS5 was used to change GAS5 expression in septic mice and HMGB1 was overexpressed in septic mice with reduced GAS5 expression. (**A**) The protein level and the phosphorylation of p65 in septic mice after GAS5 and HMGB1 intervention were measured using Western blot analysis; (**B**) effect of interference of GAS5 and HMGB1 expression on p65 nuclear translocation by immunofluorescence assay. To each group, *n*=6; data were exhibited as mean ± SD, and measured using two-way ANOVA; **, *P*<0.01, compared with the Sepsis + LV-NC group; ^##^, *P*<0.01, compared with the Sepsis + LV-GAS5-shRNA + oe-NC group.

From the above results, we found that intervention of GAS5 expression can affect the expression of HMGB1, and when we inhibited the expression of HMGB1, myocardial injury was alleviated in septic mice. In previous studies, it was found that HMGB1 could affect the injury of the body by regulating the NF-κB signaling pathway [27–29]. As shown in Figure 5A, HMGB1 could interfere with the phosphorylation level of p65 (*P*<0.01). To further explore the mechanism of HMGB1, we detected p65 in HMGB1/NF-κB pathway after GAS5 knockdown. Western blot analysis was performed to measure the level and phosphorylation of NF-κB-related proteins p65 after GAS5 interference. The total p65 level presented no significant difference, while the phosphorylation of p65 was elevated in mice with sepsis. LV-GAS5-shRNA transfection decreased the phosphorylation of p65 in mice, while LV-GAS5 transfection reversed this change, and it led to greatest phosphorylation of p65 among all groups. Immunofluorescence analysis of p65 nuclear translocation showed that p65 nuclear translocation was increased in septic mice, but p65 nuclear translocation was decreased after inhibition of GAS5 expression. On the contrary, p65 in septic mice with overexpression of GAS5 was significantly higher than that in septic mice (all *P*<0.01) (Figure 5A,B).

### PTDC partly inversed myocardial depression/injury in mice induced by overexpressed GAS5

**Figure 6:**
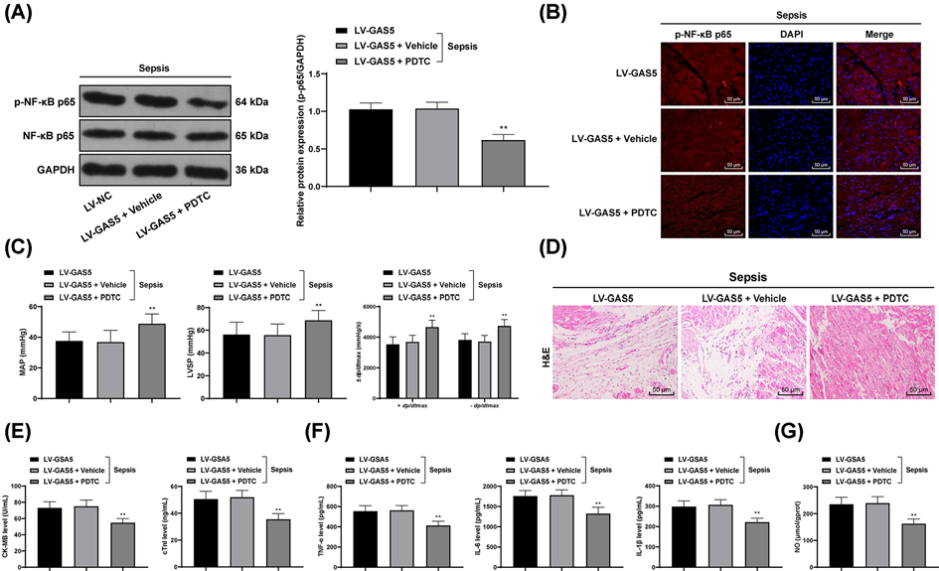
PTDC partly inversed myocardial depression/injury in mice induced by overexpressed GAS5 (**A**) Protein level p65 and the phosphorylation p65 in septic mice overexpressing GAS5 measured using Western blot analysis after PDTC treatment. (**B**) Immunofluorescence was used to detect the effect of PDTC on p65 translocation in septic mice overexpressing GAS5; (**C**) evaluation of the effect of PDTC on MAP, LVSP and ±dp/dtmax values of mice overexpressing GAS5; (**D**) the effect of PDTC on structure of myocardium of mice overexpressing GAS5 observed via HE staining; (**E**) the effect of PDTC on levels of cTnI and CK-MB in serum of mice overexpressing GAS5 measured using ELISA; (**F**) the effect of PDTC on levels of inflammatory factors TNF-α, IL-6 and IL-1β in myocardial tissues of mice overexpressing GAS5 measured using ELISA; (**G**) the effect of PDTC on NO content in myocardial tissues of mice overexpressing GAS5 evaluated using nitrite/nitrate kit. In panels; in panel (B) *n*=12, while in the rest of the panels, *n*=6; data are expressed as mean ± SD; data analyzed using one-way or two-way ANOVA and Tukey’s multiple comparisons test; **, *P*<0.01, compared with the LV-GAS5 + Vehicle group.

To further identify whether the NF-κB pathway was accountable for the myocardial depression/injury in mice, CLP-treated mice were treated with PDTC, an NF-κB inhibitor, and then the phosphorylation of p65 was decreased and p65 nuclear translocation was decreased (*P*<0.05) (Figure 6A,B). Overexpression of GAS5 exacerbated the impairment in mouse heart function in mice with sepsis, while PDTC alleviated the symptoms and led to tissue structure recovery (all *P*<0.01, Figure 6C,D). Besides, overexpression of GAS5 led to increased levels of CK-MB, cTnI in mouse serum, severe myocardial depression/injury and inflammation, and increased NO content in myocardial tissues, which were all reduced following PDTC administration (all *P*<0.05) (Figure 6E–G).

## Discussion

Treatment of sepsis and the following syndrome is remaining as a great challenge in modern world, and much of the sepsis-related morbidity and mortality is caused by severe derangements of the cardiovascular system [7]. The ceRNA network has been documented to be play significant roles in sepsis [10]. Here, our study explored the potential ceRNA network in septic mouse models and identified that lncRNA GAS5 could interact with miR-449b and HMGB1, and further promote the sepsis-induced myocardial depression with the activation of the NF-κB signaling pathway.

Initially, sepsis was induced by performing CLP in mice, after which the mouse models presented significant myocardial depression, injury and inflammation, showing as decreased MAP, LVSP and *±dp/dt*max values, and increased cTnI, CK-MB, TNF-α, IL-6, IL-1β and NO levels in mice. Sepsis usually develops following acute and systemic damage, including severe burn, trauma, and surgery, and then causes acute organ impairment and septic shock [6]. To this end, CLP has become the mostly frequently applied inducement for sepsis in rodents [24]. cTnI is a cardiac specific protein that is greatly sensitive to myocardial injury, and it secretes into the blood instantly once myocardial cell membrane is damaged [25]. Accumulation of TNF-α has been identified as one of the mechanisms involved in the inflammation-induced heart dysfunction [30]. Besides, NO plays an important role in the development of septic cardiomyopathy [31]. TNF-α, secreted in the early stage of sepsis and closely linked with cardiac systolic and diastolic impairment, induces the production of inducible NO synthase and the following NO elevation, which further leads to vasodilation and inflammation in sepsis [25].

Importantly, we found high expression of GAS5 in mice following sepsis, and its down-regulation led to alleviated myocardial depression and injury, with all the CLP-induced changes were reversed following GAS5 silencing. GAS5 has been found as a tumor inhibitor in several cancer types [32,33], however, its pro-inflammatory roles have also been identified. For instance, GAS5 has been found highly expressed in atherosclerosis and promotes arterial inflammation [34]. Besides, it has been suggested that down-regulation of GAS5 could reduce the myocardial inflammatory injury induced by palmitic acid [35]. Likewise, silencing of GAS5 has been found to improve the cardiac function, alleviate pathological injury, and decrease myocardial cell death in mice with acute myocardial infarction [36]. As we mentioned before, sepsis may be accompanied with hypotension and hypoperfusion, while high expression of GAS5 has been found to be linked with hypoxia-induced cardiomyocyte apoptosis [37]. Here, our study identified that GAS5 might hold accountability for sepsis-induced myocardial depression, injury and inflammation, since its down-regulation led to improved heart function, and decreased cTnI, CK-MB, TNF-α, IL-6, IL-1β and NO levels in mice .

Next, we further explored the potential molecules involved in the events above. The bioinformatics systems and dual luciferase reporter gene assay suggested that GAS5 could directly bind to miR-449b and further elevate the expression of HMGB1. The interactions were further confirmed through RT-qPCR. miR-449b is an uncommonly investigated miRNA with its roles largely unrevealed except its inhibiting roles in several cancer types [38,39]. As partially coincident with our findings, miR-449b-5p has been documented to alleviate hepatocyte injury following liver ischemia and reperfusion via targeting HMGB1 [40], indicating its role in injury alleviation. HMGB1 is a crucial late inflammatory regulator in the body [41]. Furthermore, overexpression of HMGB1 attenuates the protective effect of GAS5 knockdown on myocardium in septic mice. During sepsis, the alarmin HMGB1 secrets from tissues and induces systemic inflammation through interacting with multiple target cell receptors, and then results in multiorgan injury [42]. HMGB1 was found to activate the NF-κB signaling pathway to induce injury and inflammation [29,43,44]. miRNAs have been shown to directly target key proteins in the NF-κB signaling pathway, and lncRNAs contain modular domains through which they directly interact with NF-κB signaling proteins, thus regulating inflammatory response [45]. Moreover, our study identified that silencing of GAS5 further inactivated the NF-κB signaling pathway. Activation of NF-κB signaling pathway has been well-documented to play key roles in promoting sepsis and sepsis-induced inflammation and myocardial depression [18,46]. Expressions of NF-κB p65 protein, TNF-α, IL-1β, IL-6 and NO in myocardial tissues, together with levels of cTnI in serum were significantly elevated rats, while inhibition of TLR4-NF-κB signaling pathway could lead to reduced levels of downstream inflammatory factors and a protective effect on myocardial depression caused by sepsis [25]. Combining the results that the NF-κB inhibitor PDTC partially alleviated the myocardial injury induced by overexpression on GAS5, it could be inferred that the NF-κB activation might be responsible for GAS5-induced myocardial depression following sepsis.

To sum up, our study provided evidence that GAS5 might up-regulate the pro-inflammatory factor HMGB1 expression via sponging miR-449b, further activate the NF-κB signaling pathway, and promote sepsis and the following myocardial depression and injury. These findings may provide novel insights into this field that silencing of GAS5 might serve as a therapeutic option for sepsis and sepsis-induced syndrome. We hope more studies would be carried out in the near future to validate our findings and, to figure out more potential mechanisms involved in sepsis.

## Data Availability

All the data generated or analyzed during the present study are included in this published article.

## Abbreviations

ANOVA: analysis of variance
ceRNA: competing endogenous RNA
CK-MB: creatine kinase-MB
CLP: cecal ligation and puncture
cTnI: cardiac Troponin I
DAPI: 4′,6-Diamidino-2-Phenylindole
GAS5: growth arrest specific 5
HE: Hematoxylin and Eosin
HMGB1: high-mobility group box 1
IL: interleukin
lncRNA: long non-coding RNA
LV: lentiviral vector
LVSP: left ventricular systolic pressure
MAP: mean arterial pressure
miRNA: microRNA
NO: nitric oxide
PDTC: pyrrolidine dithiocarbamate
RT-qPCR: reverse transcription quantitative polymerase chain reaction
TNF-α: tumor necrosis factor-α
